# Multimodal assessment of brain stiffness variation in healthy subjects using magnetic resonance elastography and ultrasound time-harmonic elastography

**DOI:** 10.1038/s41598-024-79991-y

**Published:** 2024-11-19

**Authors:** Stefan Klemmer Chandía, Jakob Schattenfroh, Spencer T. Brinker, Heiko Tzschätzsch, Ingolf Sack, Tom Meyer

**Affiliations:** 1 Department of Radiology, Charité-Universitätsmedizin Berlin, Corporate Member of Freie Universität Berlin, Humboldt-Universität zu Berlin, Berlin Institute of Health, Charitéplatz 1, 10117 Berlin, Germany; 2 Department of Neurology, Yale School of Medicine, 333 Cedar St, New Haven, CT 06510 USA; 3 Department of Medical Informatics, Charité-Universitätsmedizin Berlin, Corporate Member of Freie Universität Berlin, Humboldt-Universität zu Berlin, Berlin Institute of Health, Invalidenstraße 90, 10115 Berlin, Germany

**Keywords:** Magnetic resonance elastography, Transtemporal ultrasound time-harmonic elastography, Human brain, Optical tracking, Fiducial markers, Biomedical engineering, Preclinical research, Brain imaging, Magnetic resonance imaging, Ultrasonography, Medical imaging, Three-dimensional imaging

## Abstract

Magnetic resonance elastography (MRE) is a noninvasive brain stiffness mapping method. Ultrasound-based transtemporal time-harmonic elastography (THE) is emerging as a cost-effective, fast alternative that has potential applications for bedside monitoring of intracranial pressure. We aim to investigate the accuracy of THE in comparison to MRE performed in the brain. Ten healthy volunteers (25–40 years old) underwent multifrequency MRE (20–35 Hz) and THE (27–56 Hz). Fiducial-marker-based optical tracking of the ultrasound field of view was used to align THE to 3D MRE. THE- and MRE-derived shear wave speed (SWS) was determined as a measure of brain stiffness and averaged within regions of various depths for cross-modality correlation analysis. MRE-measured SWS ranged from 1.0 to 1.3 m/s and was negatively correlated with age (R^2^ = 0.44, *p* = 0.035). After registration of both modalities, SWS values were linearly correlated (MRE: 1.14 ± 0.08 m/s, THE: 1.13 ± 0.10 m/s; R^2^ = 0.62, *p* = 0.007). Best agreement between modalities was achieved at depths of 40–60 mm, suggesting this range provides a viable trade-off between ultrasound attenuation and near-field bias. Similar brain regions can be consistently measured with both elastography modalities, despite the regional and individual variations of stiffness. Transtemporal THE yields stiffness values in a range similar to those obtained with more expensive MRE.

**Supplementary Information:**

The online version contains supplementary material available at 10.1038/s41598-024-79991-y.

## Introduction

Elastography is a widely used imaging technique for noninvasive, quantitative assessment of soft tissue mechanics^1^. Magnetic resonance elastography (MRE) of the human brain^2–4^ has shown to be sensitive to physiological effects^5,6^, disease-related stiffness changes^7^, and regional variation in tissue stiffness^8^ and provides highly reproducible results^9^. However, MRE requires expensive hardware, patient preparation—including transport to the scanner—and acquisition times on the order of minutes, which hinders its widespread use as a point-of-care modality in hospitals^10^.

A cost-effective, rapid, and portable alternative to MRE is ultrasound time-harmonic elastography (THE^11^). THE uses ultrasound to track harmonic displacements induced by external actuators in the same frequency range as MRE. Identically, mechanical properties of biological tissues are inferred from the local wavelengths of travelling shear waves. THE was developed and validated in phantoms^11,12^ and a range of organs including the liver^11,13,14^, spleen^11^, kidney^15,16^, pancreas^17^, skeletal muscle^18^ and aorta^19,20^. In the brain, THE holds promise as a non-invasive measurement technique for intracranial pressure as demonstrated in healthy volunteers and patients with intracranial hypertension^6,21,22^. However, transcranial sonography faces particular challenges compared with abdominal applications, e.g. abundant ultrasound reflections at skull-fluid-tissue interfaces that significantly degrade image quality^23^ and potentially distort stiffness measurements. Therefore, transtemporal THE needs validation using ground truth values obtained by, e.g., MRE. Nevertheless, there is also a need for validation of brain MRE with respect to regional and interindividual variation as demonstrated by the spread of values observed across different MRE techniques^24,25^.

Therefore, we aim to cross-validate MRE and THE by measuring the average stiffness of the same brain region, particularly the temporal lobe, since THE can image it through the temporal lobe window, and MRE-derived temporal-lobe-averaged shear wave speed (SWS) has shown a median coefficient of variation smaller than 2%^24^. However, given the heterogeneity of the brain, image registration is necessary to align both techniques. In recent years, optical tracking systems for real-time MR-guided ultrasound have gained popularity in the context of neuronavigation-guided focused ultrasound^26^. Such systems track the position and orientation of the ultrasound transducer and enable real-time localization of the THE field of view (FoV) inside a pre-acquired brain MR scan.

In our study, we used an open source optical tracking framework proposed by Preiswerk et al.^27^ to correlate THE with MRE in a group of healthy subjects. Our hypothesis was that MRE-measured SWS, as the ground truth of in vivo cerebral stiffness, is inherently variable between healthy individuals^2^. Our aim was to test the inter-individual variability of temporal lobe stiffness in healthy young subjects obtained by rapid and inexpensive THE and, if the hypothesis can be verified, to reproduce THE by MRE.

To this end, we used established multifrequency protocols for THE^22^ and MRE^8^, which had slightly shifted ranges of mechanical excitation frequencies. Our intention was to maintain the optimized vibration frequencies and waveforms used in both modalities and to present results that can be easily translated into clinical protocols.

## Materials and methods

**Fig. 1:**
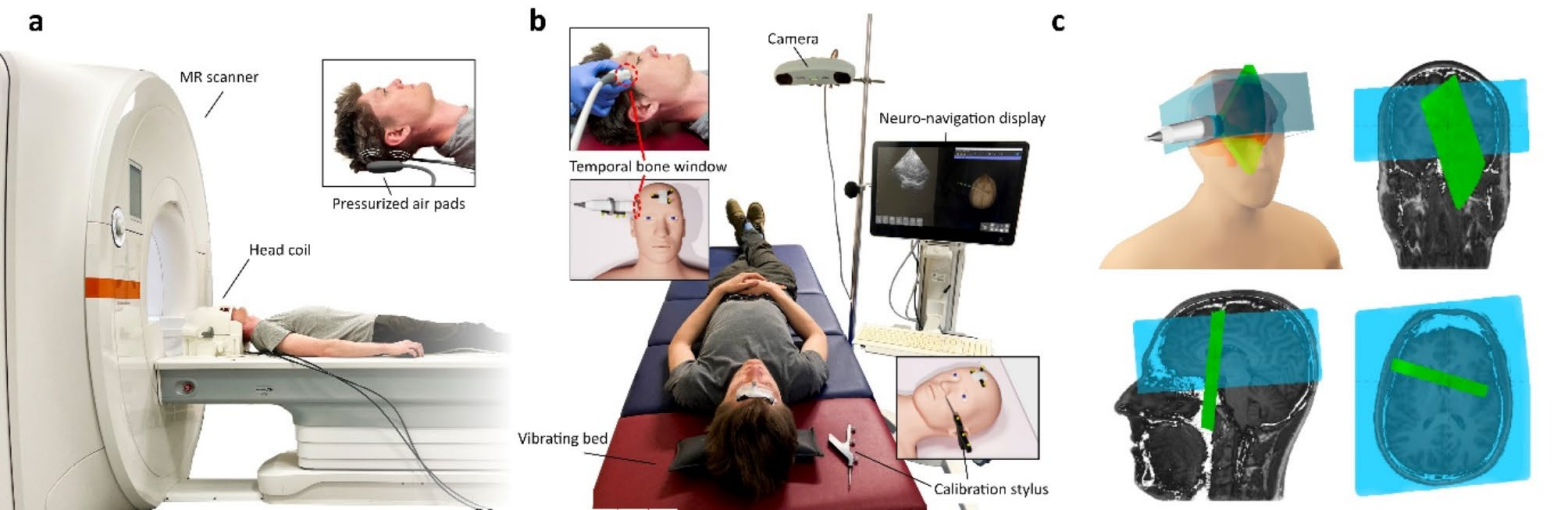
(**a**) MRE experimental setup. Two pressurized air pads placed underneath the head of the subject induce vibrations in the brain. (**b**) THE and optical tracking experimental setup. Fiducial markers (spheres) are attached to the head, a stylus, and the ultrasound probe. First, the optical tracking system is calibrated by pointing the stylus at previously chosen anatomical landmarks. Next, THE is performed by vibrating the head of the subject using a patient bed with an integrated speaker. During the THE examination, the ultrasound probe is positioned at the right temples of the subject and the exact position is recorded by the camera and projected onto the rendered and localized T1-weighted MRI map, thus enabling a neuronavigation display. (**c**) Resulting alignment of the FoVs of MRE (blue) and THE (green). 3D render (upper left corner) and 2D projections onto a T1-weighted MRI map (upper right: coronal view, lower left: sagittal view, lower right: axial view) are included. Written informed consent for publication of their clinical images was obtained from the subject. A copy of the consent form is available for review by the Editor of this journal.

This study was approved by the institutional review board of Charité – Universitätsmedizin Berlin, complies with the principles of the Declaration of Helsinki^28^, and written informed consent was obtained from all volunteers before study inclusion and from the subject depicted in Fig. 1 for publication of their clinical images. Ten volunteers (age range: 25–40 years; 9 males, 1 female) with no history of neurological disease underwent whole-brain anatomical MRI and multifrequency MRE of the brain, immediately followed by transtemporal THE.

An optical tracking procedure was used to record the site of each THE scan relative to the MRE acquisition for retrospective alignment. Finally, MRE and THE SWS maps were registered onto the Montreal Neurological Institute (MNI) atlas^29^, and regions of interest (ROIs) were automatically segmented. SWS measured in these regions was analyzed for correlations and age-related variation.

### MRE and MRI

Multifrequency MRE was performed using the same protocol described in Herthum, et al.^8^. All examinations were performed in a 3-Tesla MRI scanner (MAGNETOM Lumina, Siemens, Erlangen, Germany) equipped with a 32-channel head coil. Vibrations (20 Hz, 25 Hz, 30 Hz and 35 Hz) were consecutively induced into the brain using two pressurized air pads placed underneath the head (Fig. 1a). The 3D wave fields at the respective frequencies were encoded in 40 transverse slices through a central brain slab using a single-shot, spin-echo sequence with echo-planar imaging readout^30^. The AutoAlign tool (Siemens, Erlangen, Germany) was used to find the optimal position of the scan. For each wave propagation direction and frequency, eight equidistant time steps were encoded using a flow-compensated motion-encoding gradient with 34 mT/m amplitude. Further acquisition parameters were: 4700 ms repetition time (TR), 70 ms echo time (TE), 202 × 202 × 80 mm^3^ FoV, and 126 × 126 × 40 matrix size, resulting in 1.6 × 1.6 × 2 mm^3^ voxel size. The total acquisition time was 7:57 min. For anatomical registration, a whole-brain T1-weighted (T1w) MPRAGE sequence was acquired using the following parameters: 2300 ms TR, 2.27 ms TE, 250 × 250 × 192 mm^3^ FoV, 256 × 256 × 192 matrix size, and 0.98 × 0.98 × 1 mm^3^ voxel size, which took in total 3:54 min.

### THE

Brain THE was performed using a commercially available elastography system (GAMPTmbH, Merseburg, Germany) equipped with a phased-array transducer (P5-1S15-A6, 3 MHz center frequency). The volunteer was positioned supine on a patient bed with an integrated voice-coil actuator for vibration (GAMPTmbH, Merseburg, Germany), as described in Tzschätzsch, et al.^22^ (Fig. 1b). Continuous harmonic vibrations with six superimposed frequencies (27 Hz, 33 Hz, 39 Hz, 44 Hz, 50 Hz and 56 Hz) were induced. The ultrasound transducer was positioned at the right temple for access of the brain through the temporal bone window (see Fig. 1). The real-time optical tracking-based neuronavigation display was used for additional guidance. Radiofrequency (RF) data were acquired over 1 s acquisition time at a framerate of 80 Hz, 12 cm depth and a focus depth of 5 cm. Since the framerate imposes a Nyquist frequency limit of 40 Hz, we recovered the 44, 50 and 56 Hz frequency components through controlled aliasing at their corresponding aliased frequencies of 36, 30 and 24 Hz, respectively^11,22^. The entire scan was repeated 15 times to increase reliability.

### Optical tracking

The optical tracking procedure followed the open source framework proposed by Preiswerk et al.^27^ for noninvasive brain stimulation. A specialized camera (Polaris Vicra, Northern Digital, Inc, Ontario, Canada) tracked the position of attachable, retroreflective fiducial markers (NDI passive spheres, Northern Digital, Inc, Ontario, Canada) located on the ultrasound transducer, a stylus (Brainsight, Rogue Research Inc., Québec, Canada), and the head of the subject (Fig. 1b). The camera employs two arrays of light-emitting diodes and two sensors for sending and receiving infrared light pulses. The position of each marker relative to the camera was calculated from the angulation of the light reflected at each marker^31^ with a previously reported accuracy of 0.93 mm^27^. This information was transmitted to the PLUS toolkit^32^ in a computer (Intel Core i5-6300 CPU, 2.4 GHz, 8GB RAM) and later processed in 3D Slicer^33^ and the SlicerIGT extension^34^ to map the tracked positions onto the acquired 3D T1w MRI scan. After MRI acquisition and before THE examination, the system was calibrated by attaching fiducial markers to the head with surgical tape, and performing point-based registration of five anatomical landmarks near the eyes, nose, and left ear using a stylus^27^. Those landmarks were marked on each T1w MRI, the stylus was pointed at them, and the optical system saved their position in the camera coordinate system (Fig. 1b). After calibration, the ultrasound imaging sector was projected in real time onto the 3D-rendered T1w MRI, providing a neuronavigation display (Fig. 1b). During the scan, the positions of four calibrated reference points attached to the ultrasound transducer were stored along with the THE data to later derive the orientation and position of the THE FoV relative to the MRE data. Therefore, a coordinate transformation with nearest neighbor interpolation was performed as detailed online in the Supplementary Method.

### Shear wave speed calculation

SWS maps were generated from MRE and THE data using the same wavenumber-based multifrequency elasto-visco inversion (k-MDEV) algorithm^35^. For MRE data, tissue motion is directly encoded in the phase of the MR signal. Therefore, MRE data were unwrapped using the Laplacian method^36^, followed by temporal Fourier transformation, extraction of vibration frequencies, directional filtering with eight Gaussian filters, spatial denoising, compression wave suppression and wavelength constrain to biological limits using a bandpass filter of 15 m^− 1^ and 230 m^− 1^ high-pass and low-pass thresholds^8^, and slice by slice computation of phase gradients^8^. For THE, tissue motion was estimated based on the phase difference between complex-valued line signals according to the algorithm presented in Kasai, et al.^37^ with a window size of 32 and a stride of 8. Subsequently, the induced vibration frequencies were isolated using temporal Fourier transformation^22^. Similar to previous transtemporal THE studies, denoising was performed with bandpass filters of 37 m^− 1^ and 180 m^− 1^ high- and low-pass thresholds^6,22^. A comparison of the filtered wavefields obtained by both methods is presented online in the Supplementary Fig. 1. Finally, *k*-MDEV gradient inversion is used to obtain maps of shear wave speed. In brief, the wavefields are decomposed into plane shear waves using a directional filter with 8 filter directions. Then, wavenumbers were obtained for each frequency and filtered plane shear wave, by analyzing the local phase-gradient using first-order 2D derivatives. SWS maps were obtained by inversion of the wavenumbers and averaging of plane wave directions and frequencies^38^.

### Alignment, atlas registration, and segmentation

**Fig. 2:**
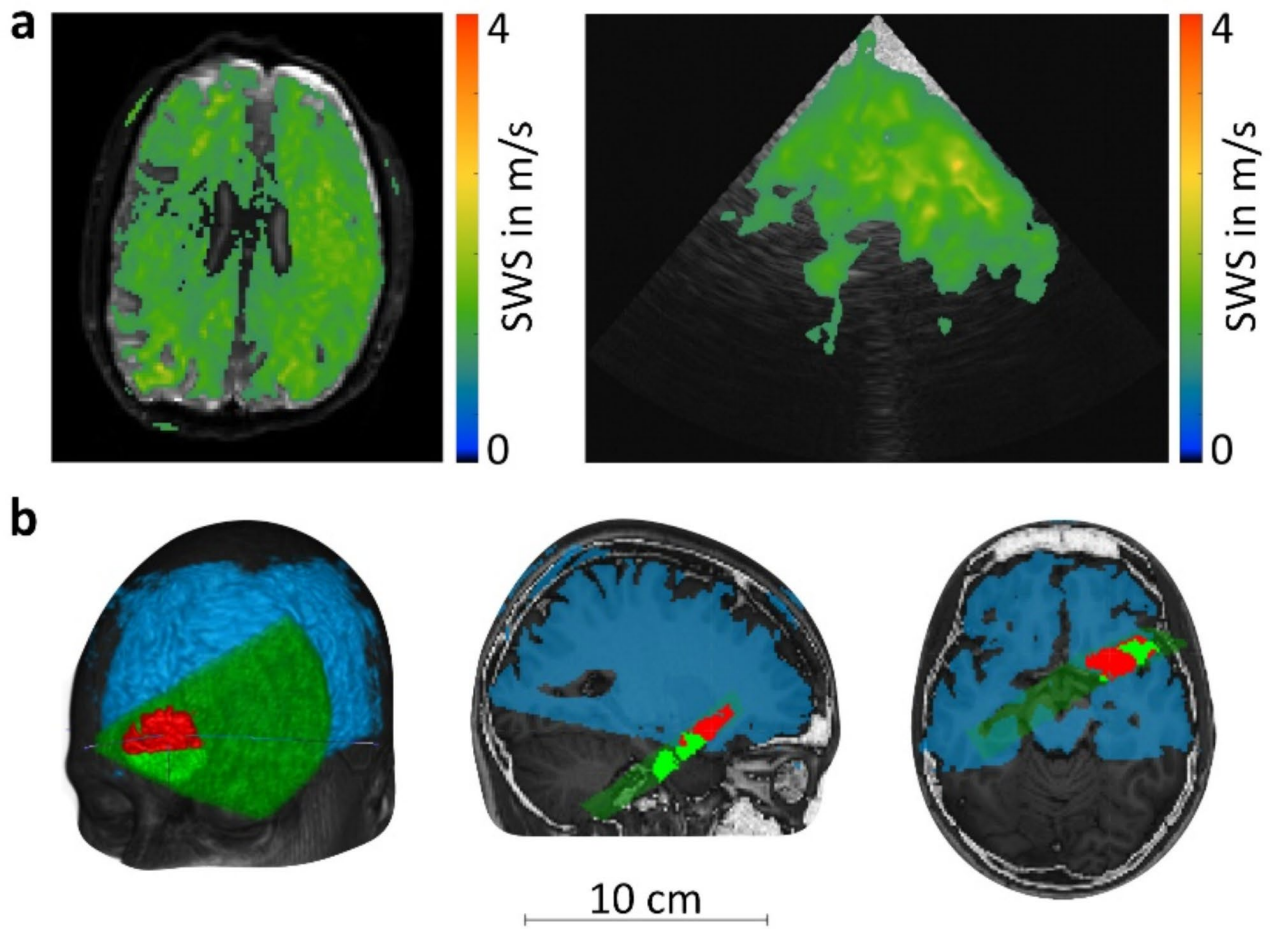
(**a**) Examples of thresholded elastograms generated from (left) MRE and (right) THE. (**b**) Three views of the temporal lobe region (red) of a subject after shear wave speed thresholding and median-averaging. This region results from the derived threshold of 0.9 m/s and the overlap of more than 8 THE scans. Gray: T1-weighted 3D MRI for anatomical orientation. Blue: thresholded MRE. Dark green: field of view of THE. Light green: thresholded THE. Red: overlap region of thresholded MRE and THE, i.e., temporal lobe region.

Once 3D MRE and 2D THE SWS maps were generated (Fig. 2a), THE maps were aligned into the MRI image space by using the stored position information from the optical tracking system. For re-gridding the ultrasound data to 3D space, a slice thickness of 12 mm was approximated based on the height of the ultrasonic elements. Next, MRE and THE SWS maps were registered to the MNI atlas^29^ using SPM12^39^. Then, the median across the 15 repeated THE scans was obtained in 3D space in a voxelwise fashion, considering each individual scan position. Voxels not covered by at least eight scans were excluded from further analysis. A ROI (Fig. 2b) of the temporal lobe—excluding sulci and cerebrospinal fluid (CSF)—was automatically segmented by setting a lower limit for both MRE and THE SWS values, as explained in the “Results” section.

### Statistical analysis

Three analyses were performed. First, the correlation between MRE and THE was investigated using linear regression and Bland-Altman analysis. By default, the comparison was carried out in 3D MRE space. Thus, every 2D THE acquisition was positioned in 3D MRE space using the optical tracking information. Alternatively, the comparison was carried out in 2D THE space. Therefore, a single slice from the 3D MRE space was extracted based on the position and orientation of the first THE acquisition. Here, the median SWS among all THE scans was calculated in a pixelwise fashion directly in 2D space, without consideration of each individual scan position. The goal of the alternative procedure was to estimate the effect of regional variability and misalignment. For brains without regional variability, both analyses should yield similar results.

Second, the temporal lobe ROI was segmented into subregions based on their distance from the temporal bone window to the center of the brain (henceforth called depth-resolved analysis). Here, four sub-ROIs were selected at 20 to 60 mm depth in increments of 10 mm, providing four MRE- and THE-derived SWS values for each subject. These were analyzed using paired Wilcoxon tests and linear regression analysis for each depth. A series of statistical tests, as proposed by Diedenhofen and Musch^40^, were conducted to identify any differences between the correlation coefficients at different depths, treating them as two non-overlapping datasets with dependent variables. Third, a correlation analysis between temporal-lobe-averaged SWS values and age was performed using linear regression analysis. In general, the linear regression equations and their coefficient of determination were reported and P-values smaller than 0.05 were considered statistically significant.

## Results

**Fig. 3:**
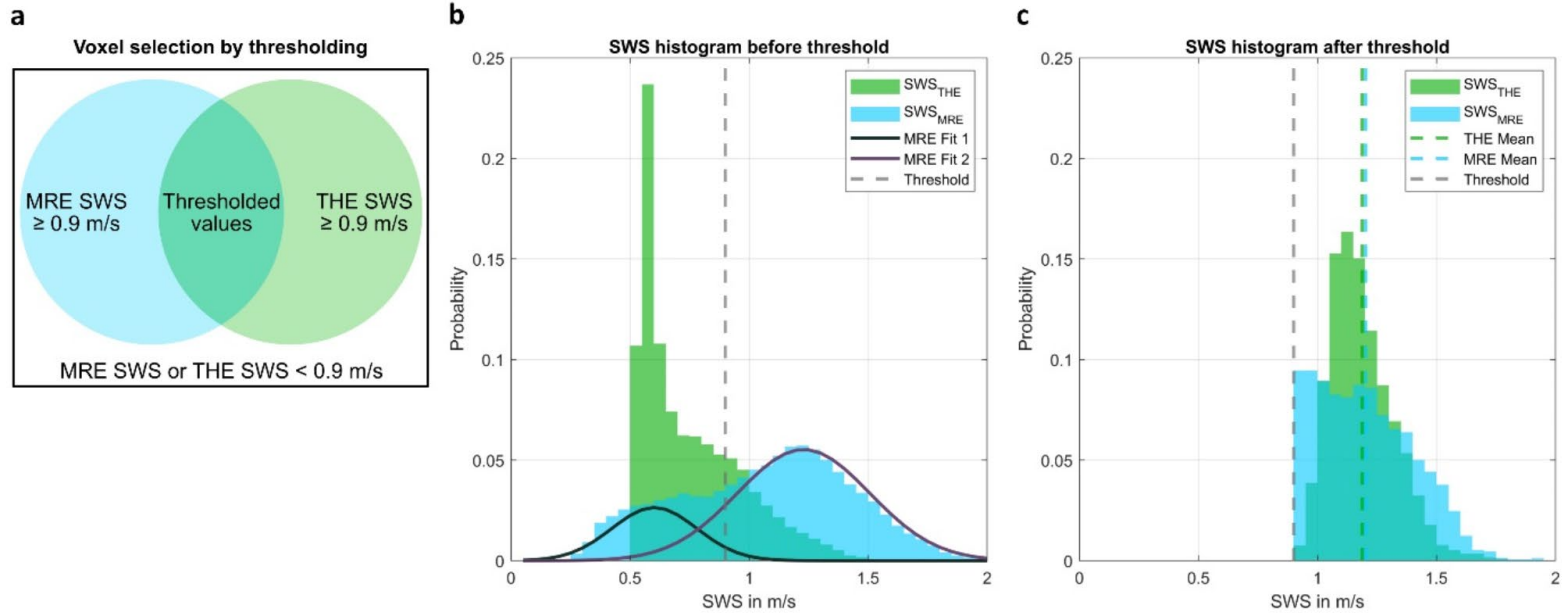
(**a**) Venn diagram depicting the selection of thresholded shear wave speed (SWS) values based on the voxels where MRE and THE are simultaneously greater than 0.9 m/s. Histogram of MRE and THE SWS values (**b**) before and (**c**) after applying the threshold to exclude values related to fluid compartments, slip interfaces, and noise. In (**b**), the bimodal Gaussian fit of MRE SWS values, which was used to derive the threshold of 0.9 m/s, is shown (gray dashed, vertical line). In (**c**), the resulting mean SWS values derived from THE and MRE are indicated by green and light-blue vertical dashed lines.

MRE, THE and the optical tracking procedure were successfully performed on all 10 healthy subjects. The overlap of MRE and THE maps in the 3D T1w MRI space of a volunteer is illustrated in Fig. 2b. Consistent with previous MRE^8^ and THE^22^ brain studies, cerebral tissue was separated from CSF by applying a lower bound to the SWS values of both methods (Fig. 3a), yielding the temporal lobe ROI of each subject. The specific SWS threshold was determined using the histogram of the voxelwise pooled MRE SWS values from all subjects inside their respective overlapping regions (Fig. 3b). A bimodal Gaussian distribution was observed, indicating the presence of distinct compartments with lower SWS values (fluid, noise) and higher SWS values (cerebral tissue). Therefore, a bi-Gaussian function with two amplitudes, mean values, and standard deviations as free variables was fit to the histogram. The resulting higher and lower Gaussian SWS distributions (mean ± standard deviation) were 0.60 ± 0.06 m/s and 1.22 ± 0.15 m/s, respectively. The threshold was determined as the central value between the two means, i.e. 0.9 m/s. After thresholding, average values of 1.20 ± 0.20 m/s and 1.19 ± 0.14 m/s were obtained for MRE and THE, respectively (Fig. 3c). An example of the resulting ROI is shown in red in Fig. 2b. The median size of the ROI across all subjects was 195 voxels (interquartile range (IQR): 97–326 voxels). The median proportion of the thresholded region size to the entire THE FoV was 19.5% (IQR: 13.2–26.2%). Among them, the median proportion of voxels with at least eight scans was 6.3% (IQR: 1.9–16.8%).

**Fig. 4:**
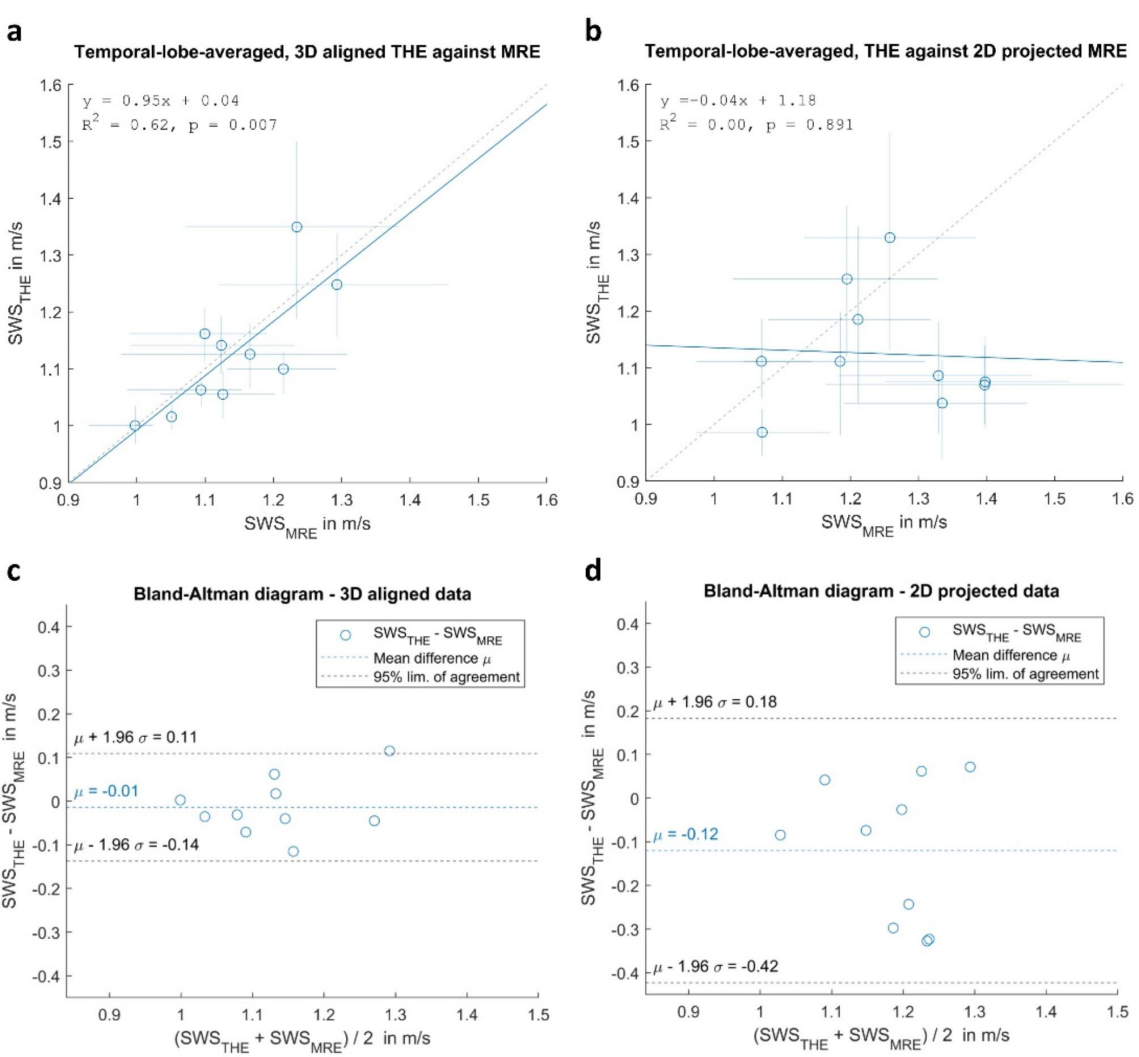
(**a**,**b**) Shear wave speed (SWS) values derived from THE against MRE, averaged over the temporal lobe region. Markers indicate mean values and lines indicate the interquartile range. Linear fits are demarcated by continuous lines. Fit equations and coefficients of determination are provided in the top left corner. (**c**,**d**) Bland-Altman plots of differences between aligned THE and MRE measurements. The mean difference is shown as a horizontal, dotted blue line, while the 95% limits of agreement are plotted in dotted gray lines. In (**a**) and (**c**), optical tracking information was used for registering THE into the 3D MRE space, whereas in (**b**) and (**d**), the comparison was carried out directly in 2D space, disregarding the positions and orientations of the individual acquisition sites. The latter method results in greater SWS variability, suggesting the presence of spatial heterogeneity.

Temporal-lobe-averaged SWS values inside the ROI were highly correlated for the 3D spatially aligned MRE and THE data (Fig. 4a), as indicated by the linear fit equation: SWS_THE_=0.95·SWS_MRE_+0.04 m/s (R^2^ = 0.62, fit p-value = 0.007). Thus, both modalities provided similar group mean SWS values (MRE:1.14 ± 0.08 m/s, THE:1.13 ± 0.10 m/s, Wilcoxon test p-value = 0.563). These group mean values differ from the mean values shown in Fig. 3b, because the former were obtained by first individually averaging the voxels in the temporal lobe ROI of each subject and then taking the mean of these averages, whereas the latter were obtained by directly averaging over all pooled voxels underlying the histograms. Bland-Altman analysis revealed 95% limits of agreement of -0.14 and 0.11 m/s with a mean difference between THE and MRE SWS of -0.01 m/s (Fig. 4c). MRE-THE differences increased with higher SWS values; however, without statistical significance in our underpowered study. Following the rationale explained in the “statistical analysis” section, an alternative analysis was carried out by taking a single slice of MRE data and directly comparing it to the median SWS across all 2D THE maps, regardless of their individual positions. As a result, THE and MRE (MRE:1.25 ± 0.2 m/s, THE:1.25 ± 0.1 m/s, Wilcoxon test p-value = 0.06) showed no correlation (SWS_THE_=-0.04·SWS_MRE_+1.18 m/s, fit p-value = 0.891), as shown in Fig. 4b and d.

**Fig. 5:**
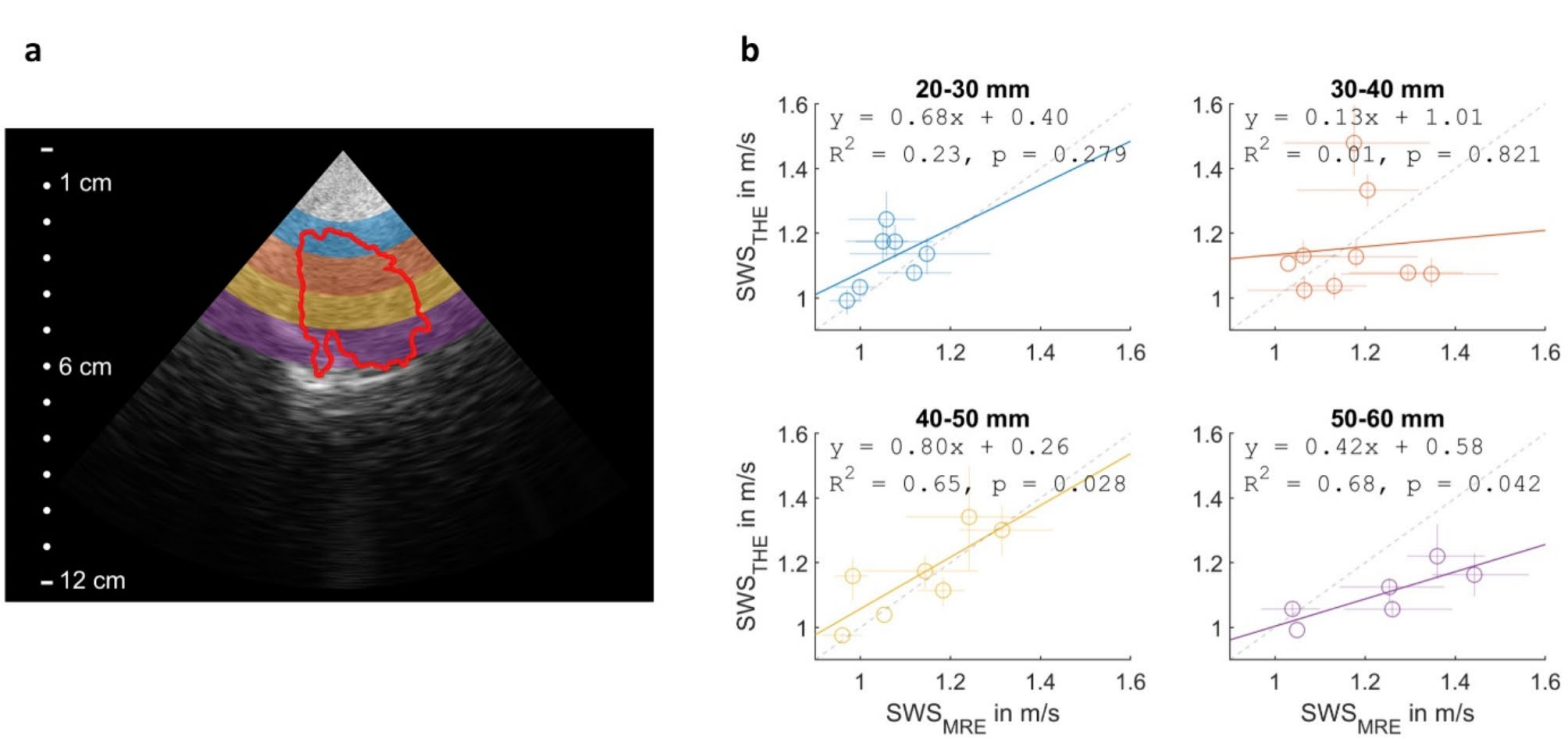
(**a**) Visualization of a THE field of view with an overlaid temporal lobe region (red) and the four depth ranges (blue, orange, yellow, and purple) used in the depth-resolved analysis. The vertical axis on the left indicates depth towards the center of the brain. (**b**) SWS values averaged inside four different depth ranges with increasing depths as given in the titles above the graphs. Markers indicate mean values and lines indicate the interquartile range. Least squares fit lines, their equations, coefficients of determination, and p-values are presented as legends.

A visual display of the depth-resolved analysis and the resulting correlations are shown in Fig. 5. Notice that some temporal lobe ROIs did not contain voxels inside all depth ranges, hence resulting in a different number of subjects for each range (Table 1). All SWS values and group correlations are summarized in Table 1. Significant MRE-THE correlations at depths of 40–50 mm (R^2^ = 0.65, fit p-value = 0.028) and 50–60 mm (R^2^ = 0.68, fit p-value = 0.042) indicate the best agreement between transtemporal THE and ground truth MRE in this range. No significant differences were observed between the correlation coefficients at 40–50 mm and 50–60 mm.

**Table 1:** Mean shear wave speed values, standard deviations, and p-values by region analyzed. The rightmost column lists reference values for the statistical tests, i.e., the condition for which the test is considered successful.

Region	Temporal lobe 0–120 mm (*N* = 10)	20–30 mm (*N* = 7)	30–40 mm (*N* = 9)	40–50 mm (*N* = 7)	50–60 mm (*N* = 6)	Reference
MRE mean SWS in m/s	1.14 ± 0.08	1.06 ± 0.06	1.17 ± 0.10	1.13 ± 0.12	1.23 ± 0.15	-
USE mean SWS in m/s	1.13 ± 0.10	1.12 ± 0.08	1.15 ± 0.14	1.16 ± 0.12	1.10 ± 0.08	-
Wilcoxon’s test p-value	0.492	0.156	1	0.375	0.063	> 0.05
THE over MRE regression p-value	0.007	0.279	0.821	0.028	0.042	< 0.05

**Fig. 6:**
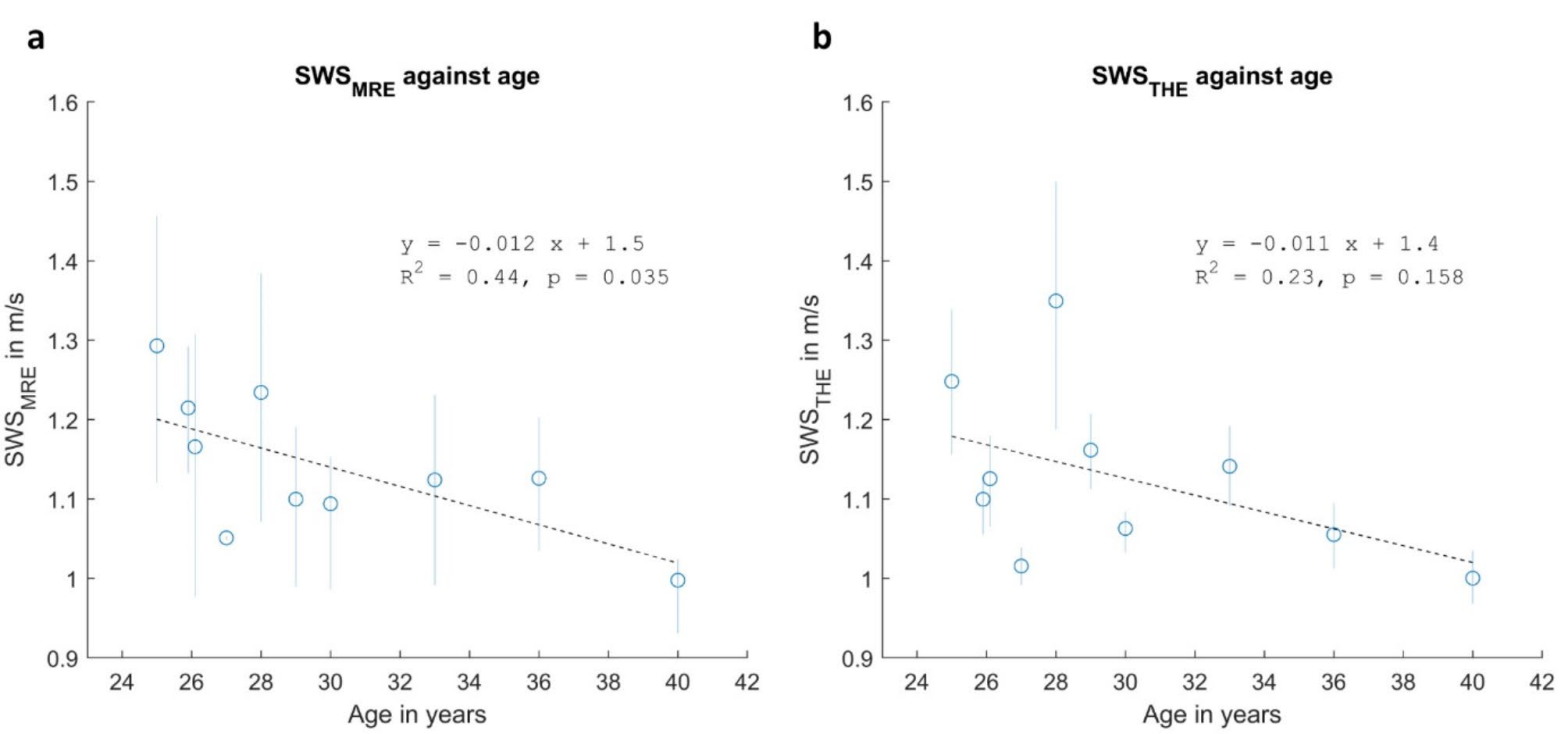
Stiffness-age correlations based on temporal lobe shear wave speed values measured by (**a**) MRE and (**b**) THE. Markers indicate mean values, while vertical lines indicate interquartile range. Linear fit is indicated as a dotted line, while its equation, coefficient of determination, and p-values are presented as legends.

To test age-related brain softening in our cohort, we further analyzed the correlation between SWS and age. Consistent with published data^41–48^, Fig. 6 shows a significant decrease in MRE values with age (R^2^ = 0.44, fit p-value = 0.035), while THE showed the same trend without reaching significance (fit p-value = 0.158).

## Discussion

To the best of our knowledge, our study is the first validation of in vivo transtemporal ultrasound-based elastography with cerebral MRE as a ground truth method. Using precise optical-tracking-based spatial alignment of the two modalities, our study showed high SWS correlation within the temporal lobe measured by MRE and THE (R^2^ = 0.62, fit p-value = 0.007). The discrepancy between the two methods, quantified by 95% limits of agreement between -0.14 and 0.11 m/s, is smaller than the ranges of clinically relevant, disease-related stiffness changes, which have been reported in the order of 0.3 m/s for intracranial hypertension^21^ or 0.5 m/s for Alzheimer’s disease^7^.

Moreover, brain stiffness was found to vary among regions and individuals, as supported by two observations. First, brain stiffness agreed between MRE and THE only after optical-tracking-based alignment in 3D space, but not in 2D space, where individual orientations and positions of each THE scan were disregarded. Therefore, without spatial alignment, THE and MRE provided SWS values from slightly different areas that did not correlate across modalities. Second, there was also heterogeneity of values across individuals in our study population, which may have been caused by individual physiological factors, e.g. age. Remarkably, the observation of different temporal lobe SWS values between subjects using MRE could be reproduced after the subjects walked from the MRI scanner to the THE bed, were placed on a different actuator system, and examined with a completely different elastographic modality.

Table 2 summarizes the results of several studies investigating the impact of aging on brain stiffness. The variation in values is attributable to numerous factors including differences in the studied brain regions, the used vibration frequencies, and the subject age range. All studies conducted so far consistently indicate that the human brain softens with age at rates between 6.5 and 15 Pa/year^41–49^. Converting our SWS values to units of Pascal using SWS^2^·ρ (with density ρ = 1000 kg/m^3^) yields a greater rate of aging-related change of 27.4 ± 11.0 Pa/year. This larger effect size may be attributed to a narrower age range with larger individual stiffness fluctuations, the use of lower vibration frequencies, or the selection of different brain slices or regions, as pointed out by Sack, et al.^42^ and Hiscox et al.^48^.

**Table 2:** Published MRE studies on the effect of aging on brain stiffness.

	*N*	Decrease in Pa per year	Value range in kPa	Investigated region	Vibration frequency in Hz	Age range in years
Sack, et al.^40^*	55	8.4	1.10–2.72	Central cerebrum slab	25, 37.5, 50, 62.5	18–88
Sack, et al.^41^*	66	10.2	1.96–4.33	24 mm thick cerebrum slab	25, 37.5, 50, 62.5	18–72
Arani, et al.^42^	45	14.0 ± 2.0	2.44–3.22	Temporal lobe	60	56–89
Kalra, et al.^43^	28	8.2	0.69–1.55	Gray matter	60	18–62
Takamura, et al.^44^	50	6.5 ± 1.3	2.28–3.00	Temporal lobe	60	20–69
		9.3 ± 1.5	1.90–2.70	Frontal lobe		
Lv, et al.^45^*	46	7.8 ± 1.3	1.65–2.44	Whole parenchyma	40, 60, 80, 90	26–76
		7.8 ± 1.3	1.63–2.37	Cortical gray matter		
Delgorio, et al.^46^	54	11.8 ± 1.3	1.87–4.55	Hippocampus	50	23–81
McIlvain, et al.^48^	125	15.0	2.14–3.96	Temporal lobe	50	5–35
This work*	10	27.4 ± 11.0	1.00–1.67	Temporal lobe	20, 25, 30, 35	25–40

*To obtain the decrease in shear modulus, we took the mean of all frequency-resolved storage moduli.

It is a strength of our study that we analyzed for the first time SWS thresholds to separate solid tissues from very low stiffness regions, which are typically considered slip interfaces or fluid compartments. The thresholding of SWS values has become a standard procedure in both MRE and THE to minimize the biased tissue values introduced by CSF and boundary artifacts^8,22^. Typically, MRE masks are defined based on anatomical T1- or T2-weighted MRI scans, followed by further refinement through SWS thresholding. The rationale behind this approach is the over-exaggeration of slip interfaces such as brain sulci by finite gradient operators in direct inversion or k-MDEV^35^. While MRE and THE typically use SWS thresholds in the range of 0.5–1 m/s, here we consistently found a value of 0.9 m/s for both MRE and THE. Consequently, these values can be used in both modalities in the future.

Our depth-resolved analysis revealed the best agreement between MRE and THE at depths of 40 to 60 mm, suggesting that THE values match the MRE ground truth within a relatively small depth window. This corresponds to a range around the ultrasound focus point, which was set for the imaging sequence and yields an optimal balance between near-field effects and ultrasound attenuation^50^.

Moreover, the effectiveness of THE for measuring tissue properties depends on its framerate and vibration waveform, i.e. frequency components, amplitudes, and phases. In previous work^22^, the vibration waveform was tuned for optimal wave amplitudes at all vibration frequencies in a region similar to the hereby identified optimal depth range. Developing adjustments to the THE vibration unit at our institution may allow THE to reach deeper tissue and hence increase the size of the overlapping region in the temporal lobe. Furthermore, in the context of pediatric applications, THE through the fontanelle could benefit from an improved image quality, which may allow for a more comprehensive assessment of mechanical properties. Such studies are currently being prepared by our group.

This study has limitations. First, the FoV of both modalities were not optimally selected, because the lower half of the THE FoV was repeatedly outside that of MRE (Fig. 1c). This considerably reduced the number of voxels eligible for comparison and hindered a more thorough and regionally resolved analysis. Nonetheless, the THE and MRE FoV configurations were selected as they are the standard setups used in previous brain elastography studies^6,8,9,22,41^. Our focus on standard setups also affected the vibration frequency match between MRE and THE, whose mean frequencies at 27.5 Hz and 41.5 Hz, respectively, differed by a factor of 1.5. However, the expected SWS dispersion in that frequency range is smaller than 0.2 m/s^51^. Additionally, optimized bandpass filters for both methods ensured that very similar group SWS values were obtained (MRE: 1.14 ± 0.08 m/s, THE: 1.13 ± 0.10 m/s), supporting the use of previously validated brain setups^8,22^. Second, reproducibility was not assessed specifically for this study. However previous studies have reported excellent test-retest reproducibility for MRE and THE of the brain as reflected in high intraclass correlation coefficients (ICC) of 0.92^22^ and 0.95^8^ for THE and MRE, respectively. Furthermore, our obtained SWS values agree with previous studies for both THE^21,22,52,53^ and MRE^41–49^, as also reflected in Table 2. Third, our study was underpowered to provide a large enough set of reference values for THE or to examine sex differences in brain properties. Nevertheless, the results obtained for spatially aligned THE and MRE as well as for aging are statistically significant and shed light on the wide heterogeneity of brain stiffness. Finally, the need for optical tracking can be avoided if THE is performed with a 3D ultrasound system, because 3D ultrasound images can be more easily aligned with 3D MRI using image registration algorithms. Nonetheless, 3D ultrasound shear wave elastography is limited in terms of framerate, penetration depth, and the need for an expensive high channel-count ultrasound system^54^. Recent developments in 2D row-column-addressing arrays may allow the implementation of 3D THE on conventional ultrasound systems, but still needs clinical validation^55^.

## Conclusion

In our study, we demonstrated the use of optical tracking for aligning brain elastography data obtained with MRE and ultrasound-based THE. Very similar in vivo stiffness values were obtained when identical regions of the human brain were investigated. In contrast, without proper spatial alignment, the difference between MRE and THE was greater than the expected heterogeneity of stiffness values due to individual effects such as aging. These findings suggest that THE can resolve the spatial heterogeneity of brain stiffness detected by MRE and may serve as a cost-effective tool to monitor changes in brain stiffness in a clinical context, such as non-invasive detection of intercranial hypertension, in the future.

## Electronic supplementary material

Below is the link to the electronic supplementary material.

Supplementary Material 1

Supplementary Material 2

## Data Availability

The data that supports the findings of this study cannot be made publicly available upon publication, because it contains sensitive personal information. However, is available upon reasonable request from the authors. For this matter, please contact corresponding author Tom Meyer (tom.meyer@charite.de).
