# Supplementary figures and images for: Multimodal assessment of brain stiffness variation in healthy subjects using magnetic resonance elastography and ultrasound time-harmonic elastography
Source: Sci Rep. 2024 Nov 19;14:28580. doi: 10.1038/s41598-024-79991-y (PMC11576992; doi:10.1038/s41598-024-79991-y)

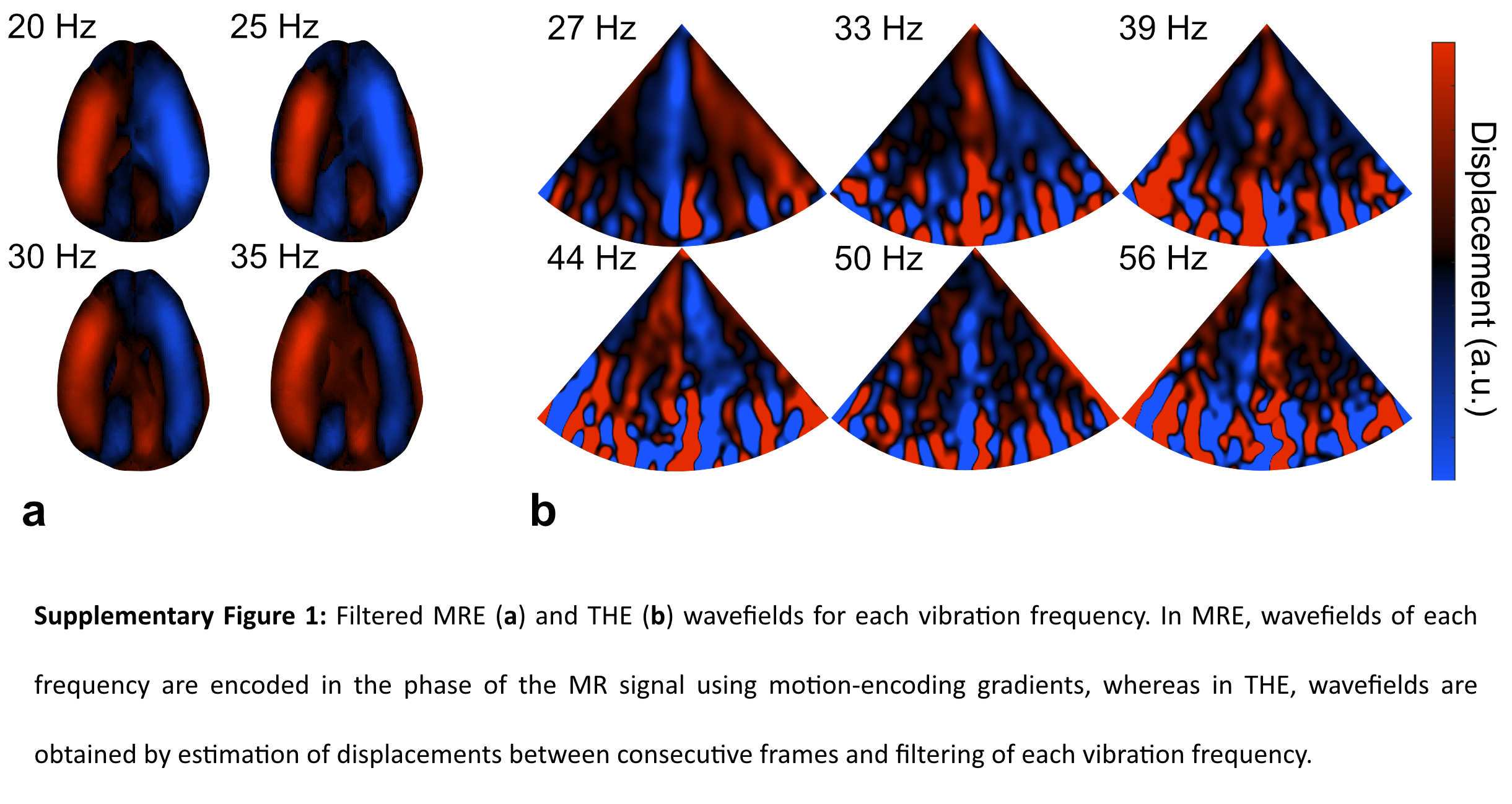

Supplement: Supplementary file 1 — Supplementary Material 1 [file 41598_2024_79991_MOESM1_ESM.jpg]
