# Supplementary material for: Multimodal assessment of brain stiffness variation in healthy subjects using magnetic resonance elastography and ultrasound time-harmonic elastography
Source: Sci Rep. 2024 Nov 19;14:28580. doi: 10.1038/s41598-024-79991-y (PMC11576992; doi:10.1038/s41598-024-79991-y)
Supplement: Supplementary file 2 — Supplementary Material 2 [file 41598_2024_79991_MOESM2_ESM.pdf]

Supplementary Method: Mapping THE stiffness maps from 2D to 3D

Stefan Klemmer-Chandía[1], Jakob Schattenfroh[1], Spencer T. Brinker[2], Heiko Tzschätzsch[3], Ingolf Sack[1], Tom Meyer[1]*

[1] Department of Radiology, Charité - Universitätsmedizin Berlin, Corporate Member of Freie Universität Berlin, Humboldt-Universität zu Berlin, and Berlin Institute of Health, Charitéplatz 1, 10117 Berlin, Germany

[2] Department of Neurology, Yale School of Medicine, 333 Cedar St., New Haven, CT 06510, United States

[3] Department of Medical Informatics, Charité - Universitätsmedizin Berlin, Corporate Member of Freie Universität Berlin, Humboldt-Universität zu Berlin, and Berlin Institute of Health, Invalidenstraße 90, 10115 Berlin, Germany

* Correspondence address

Tom Meyer

Department of Radiology

Charité - Universitätsmedizin Berlin

Charitéplatz 1

10117 Berlin, Germany

tom.meyer@charite.de

+49 30 450 539865

The aim of this study is to compare brain stiffness measurements performed on 10 subjects using both THE and MRE. However, the resulting stiffness maps from both modalities are 2D and 3D, respectively, thus THE only provides a single slice that is not aligned with MRI coordinate system. Hence, their comparison is not straightforward. To overcome this limitation, we implemented a fiducial-marker-based, optical tracking system to obtain the position and orientation of the ultrasound probe in 3D space. Thus, taking the ultrasound acquisition parameters into consideration, we were able to map the 2D slice into 3D space with defined thickness in the same coordinate system as the previously obtained 3D T1w MRI map. A diagram of this procedure is shown in figure S.1.

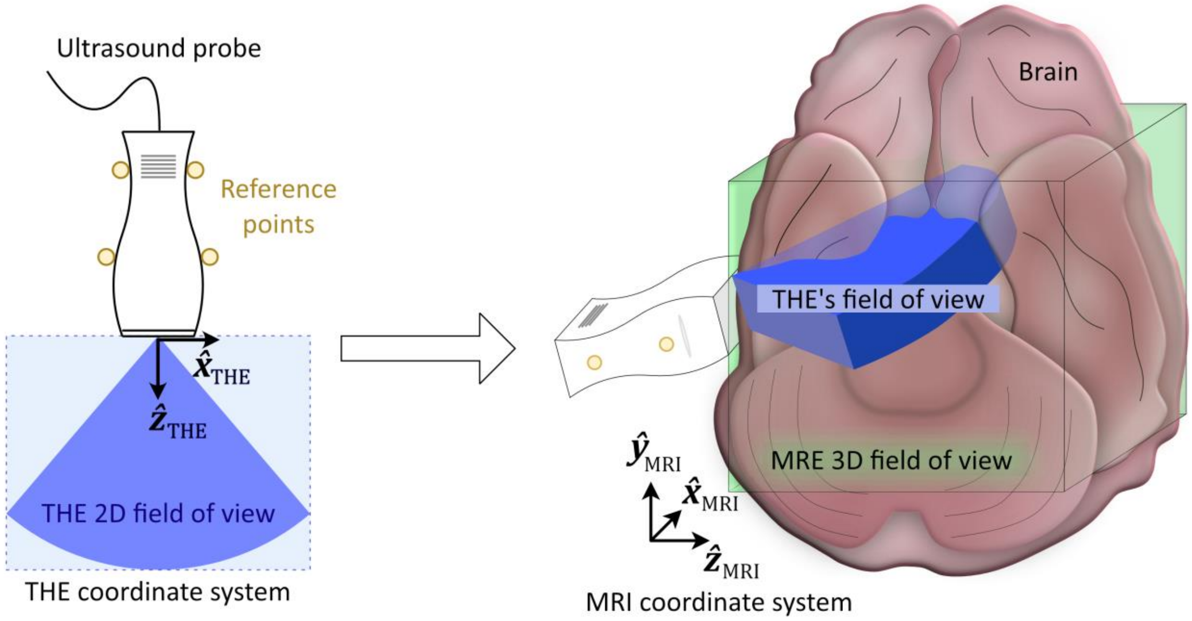

Figure S.1: Mapping procedure from 2D THE stiffness maps into 3D MRE space. The drawing was created using Affinity Designer v.1.10 (affinity.serif.com/de/designer/).

The optical tracking procedure returned the position of four reference points on the ultrasound probe as three dimensional vectors within the coordinate system of the MRI acquisition. All four points lied on the same plane. Two points were selected on the front ($\boldsymbol{r}_{\mathrm{FL}}$,

$\boldsymbol{r}_{\mathrm{FR}}$) and two on the back ($\boldsymbol{r}_{\mathrm{BL}}$, $\boldsymbol{r}_{\mathrm{BR}}$) of the probe, on the left and right side respectively, as depicted in figure S.2.

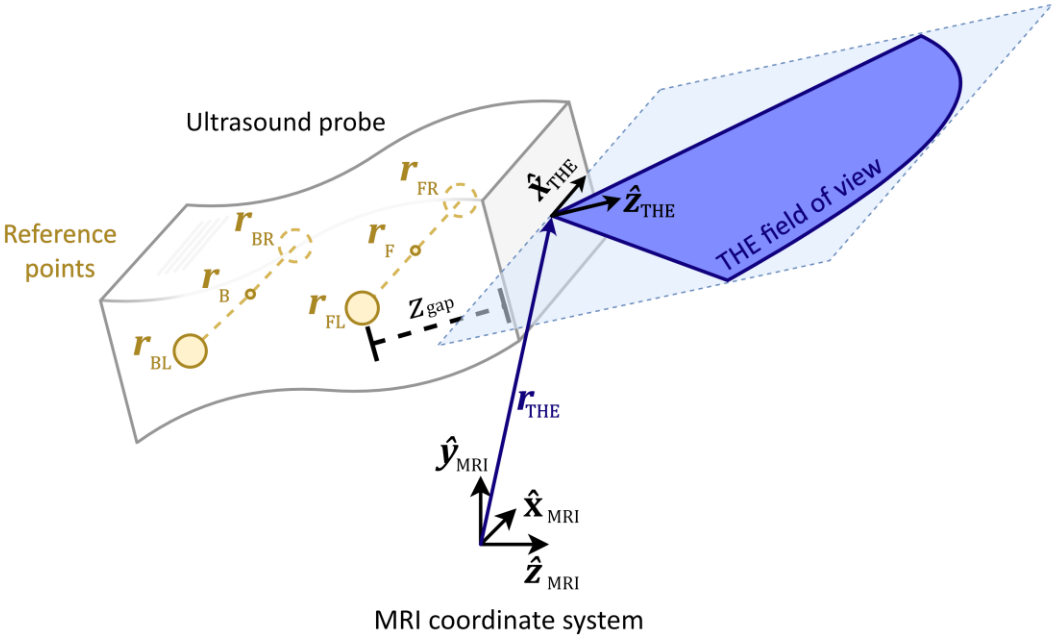

Figure S.2: Positioning of the reference points in the ultrasound probe and their coordinate system.

First, we obtained the unit vectors of the THE coordinate system in the axial ($\hat{\boldsymbol{z}}_{\mathrm{THE}}$) and lateral ($\hat{\boldsymbol{x}}_{\mathrm{THE}}$) dimensions,

$$\hat{\boldsymbol{z}}_{\mathrm{THE}} = \frac{\boldsymbol{r}_{\mathrm{F}} - \boldsymbol{r}_{\mathrm{B}}}{\|\boldsymbol{r}_{\mathrm{F}} - \boldsymbol{r}_{\mathrm{B}}\|}, \qquad \hat{\boldsymbol{x}}_{\mathrm{THE}} = \frac{\boldsymbol{r}_{\mathrm{FR}} - \boldsymbol{r}_{\mathrm{FL}}}{\|\boldsymbol{r}_{\mathrm{FR}} - \boldsymbol{r}_{\mathrm{FL}}\|}$$

Where

$$\boldsymbol{r}_{\mathrm{F}} = \frac{1}{2}(\boldsymbol{r}_{\mathrm{FL}} + \boldsymbol{r}_{\mathrm{FR}}), \qquad \boldsymbol{r}_{\mathrm{B}} = \frac{1}{2}(\boldsymbol{r}_{\mathrm{BL}} + \boldsymbol{r}_{\mathrm{BR}})$$

This allows us to obtain the position of the center ultrasonic element $\boldsymbol{r}_{\mathrm{THE}}$.

$$\boldsymbol{r}_{\mathrm{THE}} = \boldsymbol{r}_{\mathrm{F}} + z_{\mathrm{gap}}\,\hat{\boldsymbol{z}}_{\mathrm{THE}},$$

Where $z_{\text{gap}} = 18$ mm is the distance between $\boldsymbol{r}_{\text{F}}$ and the ultrasonic elements in the $\hat{\boldsymbol{z}}_{\text{THE}}$ direction.

From these vectors, the rotation matrix or the orientation of the ultrasound plane can be obtained:

$$M_{rot} = [\hat{\boldsymbol{x}}_{\text{THE}} \quad \hat{\boldsymbol{z}}_{\text{THE}} \quad (\hat{\boldsymbol{x}}_{\text{THE}} \times \hat{\boldsymbol{z}}_{\text{THE}})\ ]$$

Together with the translation given by $\boldsymbol{r}_{\text{THE}}$, we can apply the coordinate transformation between THE and MRI coordinate space as an affine transformation using MATLAB function Interp3 (The MathWorks, Inc., Natick, Massachusetts, United States) with nearest neighbor interpolation. THE coordinate system is originally positioned on the top left corner of the image. Hence, before interpolation, we apply one last translation to it by $\frac{x_{\text{FoV}}}{2}\,\hat{\boldsymbol{x}}_{\text{THE}}$, where $x_{\text{FoV}} = 234$ mm is the lateral THE field of view. Here, we consider both THE and MRI resolutions to correctly scale the input and output dimensions. Additionally, we set a slice thickness of 12 mm based on the height of the ultrasonic elements.
